# Understanding the regulatory mechanisms of endometrial cells on activities of endometrial mesenchymal stem-like cells during menstruation

**DOI:** 10.1186/s13287-020-01750-3

**Published:** 2020-06-17

**Authors:** Shan Xu, Rachel W. S. Chan, Tianqi Li, Ernest H. Y. Ng, William S. B. Yeung

**Affiliations:** 1 Department of Obstetrics and Gynaecology, Second Affiliated Hospital of Xi’an Jiaotong University, Xi’an, Shaanxi China; 2 Department of Obstetrics and Gynaecology, LKS Faculty of Medicine, The University of Hong Kong, Pokfulam, Hong Kong, SAR China; 3 Shenzhen Key Laboratory of Fertility Regulation, Reproductive Medicine Centre, The University of Hong Kong Shenzhen Hospital, Shenzhen, Guangdong China

**Keywords:** Endometrium, Cytokines, Stem cells, Menstruation, WNT signalling, RSPO1, LGR5

## Abstract

**Background:**

The identification of endometrial stem/progenitor cells in a high turnover rate tissue suggests that a well-orchestrated underlying network controls the behaviour of these stem cells. The thickness of the endometrium can grow from 0.5–1 mm to 5–7 mm within a week indicating the need of stem cells for self-renewal and differentiation during this period. The cyclical regeneration of the endometrium suggests specific signals can activate the stem cells during or shortly after menstruation.

**Methods:**

Endometrial mesenchymal stem-like cells (eMSCs) were cocultured with endometrial epithelial or stromal cells from different phases of the menstrual cycle; the clonogenicity and the phenotypic expression of eMSC markers (CD140b and CD146) were assessed. The functional role of WNT/β-catenin signalling on eMSC was determined by western blot analysis, immunofluorescent staining, flow cytometry, quantitative real-time PCR and small interfering RNA. The cytokine levels in the conditioned medium of epithelial or stromal cells cocultured with eMSCs were evaluated by enzyme-linked immunosorbent assays.

**Results:**

Coculture of endometrial cells (epithelial or stromal) from the menstrual phase enhanced the clonogenicity and self-renewal activities of eMSCs. Such phenomenon was not observed in niche cells from the proliferative phase. Coculture with endometrial cells from the menstrual phase confirmed an increase in expression of active β-catenin in the eMSCs. Treatment with IWP-2, a WNT inhibitor, suppressed the observed effects. Anti-R-spondin-1 antibody reduced the stimulatory action of endometrial niche cells on WNT/β-catenin activation in the T cell factor/lymphoid enhancer-binding factor luciferase reporter assay. Moreover, the mRNA level and protein immunoreactivities of leucine-rich repeat-containing G-protein coupled receptor 5 were higher in eMSCs than unfractionated stromal cells. Conditioned media of endometrial niche cells cocultured with eMSCs contained increased levels of C-X-C motif ligand 1 (CXCL1), CXCL5 and interleukin 6. Treatment with these cytokines increased the clonogenic activity and phenotypic expression of eMSCs.

**Conclusions:**

Our findings indicate a role of WNT/β-catenin signalling in regulating activities of endometrial stem/progenitor cells during menstruation. Certain cytokines at menstruation can stimulate the proliferation and self-renewal activities of eMSCs. Understanding the mechanism in the regulation of eMSCs may contribute to treatments of endometrial proliferative disorders such as Asherman’s syndrome.

## Introduction

Adult stem cells are responsible for maintaining tissue homeostasis. The balance between stem cell differentiation and self-renewal is essential to support tissue regeneration [1]. The specific niche, in which the adult stem cells reside, regulates stem cell function through several mechanisms among which the best studied one is the direct contact between stem cells and their adjacent niche cells [2]. Niche support of stem cells has been reported for adult stem cells in the skin, intestine and bone marrow. Key elements of adult stem cell regulation are soluble and immobilized factors within the stem cell niche. Both intrinsic and extrinsic signals are involved in the regulatory network of the niche.

The identification of endometrial stem/progenitor cells in a tissue with high turnover rate suggests the presence of a well-orchestrated regulatory network controlling stem cells fate [3]. The thickness of the endometrium can grow from 0.5–1 mm to 5–7 mm within 1 week indicating the need of the stem cells to self-renewal and differentiate during this period [4]. The cyclic regeneration of the endometrium suggests that the environmental cues at menstruation activate the stem cells. Consistently, endometrial mesenchymal stem-like cells (eMSCs) at menstruation display greater self-renewal and proliferation abilities than those in secretory phase [5]. Therefore, the unique environment constituted by the niche cells during menstruation was investigated to determine whether the signals can support eMSC functions and to delineate the mechanisms involved.

The WNT/β-catenin signalling pathway plays a role in population expansion and self-renewal of adult stem cells in a variety of mammalian organs [6]. In mesenchymal stem cells, activation of the canonical WNT signalling pathway promotes proliferation [7] and controls cell fate [8]. We recently demonstrated that myometrial cells are a niche component of eMSCs, modulating their self-renewal activity by activation of WNT/β-catenin signalling [9]. There is an enhanced expression of active β-catenin in proliferating putative stromal stem/progenitor cells in postpartum mouse endometrium [10]. Modulation of the WNT/β-catenin pathway affects regeneration and development of the porcine endometrium [11, 12].

In humans, WNT signalling plays a role in endometrial growth and regression [13]. There are differential expression of WNT signalling associated genes in endometrial epithelial cells between pre-menopausal women and post-menopausal women [14]. Primary culture of human endometrial epithelial and stromal cells expresses WNT2, 3, 4 and 5A [15]. The activity of WNT/β-catenin signalling also changes cyclically across the menstrual cycle [16, 17]. Endometrial regeneration starts at menstruation. Therefore, it is logical that the niche at this stage should activate stem cells for endometrial repair. We hypothesized that soluble factors secreted by the endometrial niche cells at menstruation regulate the eMSCs’ activities through the WNT/β-catenin signalling pathway.

During menstruation, cytokines and chemokines are important regulators of the local environment in the uterus [18]. The endometrium expresses numerous cytokines and chemokines across the menstrual cycle in which some are involved in leukocyte migration, facilitation of endometrial apoptosis and activation of proliferation of endometrial cells. Therefore, we also hypothesize that cytokines and chemokines are potential regulators of eMSCs during menstruation.

## Material and methods

### Human tissues

Ethics approval was obtained from the Institutional Review Board of the University of Hong Kong/Hospital Authority Hong Kong West Cluster. Written consents were signed by recruited subjects after detailed counselling prior to participation of the study. Full-thickness endometrial samples were acquired from 22 women with regular menstrual cycles (median age 45.5; range 41 to 52 years) who underwent total abdominal hysterectomy for benign non-endometrial pathologies (supplementary data Table S1). They had not taken hormonal therapy for 3 months before surgery. The phase of the menstrual cycle was categorized into proliferative (*n* = 9) and secretory (*n* = 13) by experienced histopathologists based on haematoxylin-eosin-stained endometrial sections. Menstrual phase samples were collected by endometrial aspiration from 23 women with regular menstrual cycles and aged from 32 to 43 years attending the infertility clinic on days 2–3 of their menstrual cycle (median age 35; range 32 to 43 years, Additional file: Table S2).

### Single-cell suspensions of endometrial epithelial and stromal cells

The isolation procedure of endometrial cells was carried out as described [5]. The tissues were minced and digested with PBS containing collagenase type III (0.3 mg/ml, Worthington Biochemical Corporation, NJ, USA) and deoxyribonuclease type I (40 μg/ml, Worthington Biochemical Corporation) for 1 h at 37 °C. Red blood cells were removed using Ficoll-Paque (GE Healthcare, Uppsala, Sweden) density-gradient centrifugation. Leukocytes were excluded using anti-CD45 antibody-coated Dynabeads (Invitrogen, Waltham, MA, USA). Epithelial cells were separated from the stromal cells using anti-CD326 (EpCAM) antibody-coated microbeads (Miltenyi Biotec Inc., San Diego, CA, USA). Freshly isolated epithelial cells and stromal cells were used for coculture and collection of condition medium as describe below. Some of the spare purified stromal cells (6000–8000 cells/cm^2^) were plated into 100-mm petri dishes coated with fibronectin (1 mg/ml, Invitrogen) and cultured in growth medium containing 10% FBS (Invitrogen), 1% antibiotics (Invitrogen) and 2 mmol/L glutamine (Invitrogen) in DMEM/F12 (Sigma-Aldrich, St Louis, MA, USA) for 7–14 days in a humidified carbon dioxide incubator at 37 °C in 5% CO_2_. The medium was changed every 7 days until the cells reached 90% confluence.

### Magnetic bead selection for endometrial mesenchymal stem-like cells

EMSCs were isolated by sequential beading with magnetic beads coated with anti-CD140b and anti-CD146 antibodies as described [5]. The stromal cells were first successively incubated with PE-conjugated anti-CD140b antibody (10 μl/10^6^ cells, R&D Systems, Minneapolis, MN, USA) for 45 min at 4 °C and then with anti-mouse IgG1-coated microbeads (Miltenyi Biotec Inc.) for 15 min at 4 °C before they were loaded onto Miltenyi MS columns with a magnetic field to collect the CD140b^+^ cells. The stromal CD140b^+^ population was cultured in fibronectin-coated dishes containing growth medium at 37 °C in 5% CO_2_ for 7–10 days to allow detachment of the microbeads during cell expansion. They were then trypsinized and incubated with anti-CD146 antibody-coated microbeads (Miltenyi Biotec Inc.) for 15 min at 4 °C. The CD140b^+^CD146^+^ cells (eMSCs) were obtained after magnetic column separation.

### Coculture

The eMSCs at clonal density (350 cells per well) and the endometrial epithelial or stromal cells (30,000 cells) were seeded onto fibronectin-coated 6-well plates and transwell inserts (EMD Millipore, Billerica, MA, USA), respectively, and were cocultured. Monoculture (culture of eMSCs without niche cells) served as the control. All conditions were performed in duplicates or triplicates.

### Preparation of other cell types

The human oviductal epithelial E6/E7 (OE-E6/E7) cell line (passage 24–26, obtained from Dr. CYL Lee) and human foreskin fibroblast (HFF-1) cell line (passage 16–19, CRL-2429, ATCC, Manassas, VA, USA) were also cocultured with eMSCs. The eMSCs were seeded at clonal density (350 cells per well) onto fibronectin-coated 6-well plates and OE E6/E7 or HFF-1 cells were seeded at 15,000 cells per insert. New inserts containing OE E6/E7 or HFF-1 cells were replaced on day 7 of culture.

### Colony forming activity

The number of colony forming units (CFUs) was recorded on day 14 of culture. The colony forming ability was determined by the number of CFUs divided by the number of cells seeded, multiplied by 100 [5].

### Flow cytometry

The coexpression of eMSC markers, CD140b and CD146 on endometrial stromal cells after 15 days of culture was analysed using multi-colour flow cytometry as described [19]. Endometrial cells were labelled with phycoerythrin (PE)-conjugated antibody against CD140b (2.5 μg/ml, mouse IgG_1_, R & D Systems) and fluorescein isothiocyanate (FITC)-conjugated anti-CD146 antibody (1 mg/ml, mouse IgG_1_, Thermo Fisher Scientific, Waltham, MA USA) or isotype-matched controls. Flow cytometry analysis was performed using a BD Fortressa (BD Biosciences, San Jose, CA, USA) and the FlowJo software (Tree Star, Ashland, OR, USA) at the Centre for PanorOmic Sciences (CPOS) Imaging and Flow Cytometry Core, The University of Hong Kong.

### Preparation of conditioned medium

Conditioned medium (CM) was collected from endometrial cells in the menstrual phase. Freshly isolated epithelial cells or stromal cells (30,000 cells) were cultured in growth medium in 6-well plate for 1 day then washed with PBS and replaced with 3 ml of growth medium. After 2 days in culture, the CM was collected, filtered sterilized and stored at − 80 °C until experimentation.

To concentrate the secretory factors from endometrial niche cells, the epithelial or stromal cells were cultured in 1 ml of serum-free DMEM/F-12 medium. The secretory factors in the CM were concentrated (CCM) after 48 h by centrifugation at 4000*g* for 20 min at 4 °C using Amicon ultra-15 centrifugal filter devices (EMD Millipore) with a molecular weight cutoff of 10 kDa. The amount of the concentrated protein derived from one culture well was considered as one unit. Epithelial or stromal CCM (1/3-unit) was added into the growth medium for eMSC culture. The CCM collected from cell free DMEMF-12 medium was used as control.

### Western blot analysis

The cellular proteins of eMSCs were extracted with cell lysis buffer (Ambion, Grandisland, NY, USA). The proteins (5 μg) were mixed with 5X SDS loading buffer (60 mM Tris-HCl, pH 6.8, 2% SDS, 0.1% bromophenol blue, 25% glycerol and 14.4 mM β-mercaptoethanol), denatured at 95 °C for 10 min, subjected to sodium dodecyl sulphate-polyacrylamide gel electrophoresis and transferred to polyvinylidene difluoride membranes (Immobilon™-P, Milllipore). The membranes were blocked with 5% skim milk in PBS containing 0.1% Tween-20 for 30 min, incubated with primary antibodies at appropriate concentrations (Additional file: Table S3) overnight at 4 °C and stained with appropriate horseradish peroxidase-conjugated secondary antibodies (Additional file: Table S3) for 1 h at room temperature. The protein bands were visualized by the WesternBright ECL Kit (Advansta, CA, USA). The intensities of the protein bands were quantified densitometry, and the values were normalized to β-actin using the ImageJ software (US National Institutes of Health, USA).

### Quantitative real-time polymerase chain reaction

Total RNA was extracted with the Absolutely RNA RT-PCR microprep kit (Agilent Technologies, Santa Clara, CA, USA). The quality and quantity of the total RNA was checked by spectrophotometry. The RNA was reverse transcribed by the high-capacity complementary DNA reverse transcription kit (Roche Applied Science, Basel, Switzerland). Taqman probe for *RSPO1* was used (Applied Biosystems, Grand Island, NY, USA). Real-time PCR was performed with a 7500 Real-Time PCR System (Applied Biosystems) using the following parameters: 2 min at 50 °C, 10 min at 95 °C, then 40 cycles of 15 s at 95 °C and 1 min at 60 °C. The results are presented as relative gene expression compared with the internal control 18S using the 2^−ΔΔCt^ method. Determination was made in triplicate from three separate samples.

### WNT reporter assay

EMSCs at a density of 20,000–50,000 per well were seeded into a 24-well plate. They were co-transfected with 4 μg of either TOPflash or FOPflash vector and 1 μg of pRL-TK (Renilla-TK-luciferase vector, Promega, Madison, WI, USA) as a control using Lipofectamine 2000 (Invitrogen). Cells were subsequently treated with epithelial cell CCM from the menstrual phase (CCM 1/3 unit: growth medium) with or without the neutralization antibodies against RSPO1 (1 μg/ml, Abcam, Cambridge, UK) for 48 h. Rabbit IgG was the isotype control (Abcam). The cells were lysed, and the luciferase activities were measured using a GLOMAX™ 96 microplate luminometer. Firefly luciferase activity was normalized against the Renilla luciferase activity for transfection efficiency. The TOP/FOP ratio was used as a measure of T cell factor/lymphoid enhancer-binding factor (TCF/LEF) transcription.

### Inhibition of WNT signalling

EMSCs seeded at clonal density were treated with epithelial CCM from the menstrual phase (1/3 unit: growth medium) with or without IWP-2 (Sigma-Aldrich) at 1.25 μM. Growth medium supplemented with dimethyl sulfoxide was used as negative control.

### Treatment with neutralization antibodies and recombinant proteins

Neutralization antibody for RSPO1 (1 μg/ml, Abcam) was added to the epithelial CCM from the menstrual phase (1/3-unit: growth medium). Isotype antibody rabbit IgG was used as negative control. Recombinant human WNT3A (12.5, 25, 50 ng/ml, R&D Systems) and RSPO1 (50 ng/ml, R&D Systems) was supplemented to the growth medium of eMSCs seeded at clonal density for 15 days.

### Immunofluorescence staining

The unfractionated endometrial stromal cells or eMSCs (8000–10,000 cells) were resuspended in growth medium and transferred to slides coated with 3-aminopropyl-triethoxysilane using a Shadon Cytospin Centrifuge (Thermo Electron, Waltham, USA) with centrifugation at 7500 rpm for 10 min followed by fixation in 4% paraformaldehyde for 20 min. Permeabilization was performed using 0.1% Triton-X 100 for 10 min and blocked with the corresponding serum for 30 min at room temperature. The slides were then incubated with the primary antibody (Additional file: Table S4) overnight at 4 °C, incubated with the secondary Alexa fluor donkey anti-rabbit 568 antibody (Thermo Scientific) for 1 h at room temperature. The cell nuclei were detected by DAPI (Thermo Scientific). Images were captured with a LSM 700 inverted confocal microscope and a LSM ZEN 2010 software (Carl Zeiss, Munich, Germany) at the Centre for PanorOmic Sciences (CPOS) imaging and Flow Cytometry Core, The University of Hong Kong.

### Cytokine array and ELISA

Cytokine Array C3 (RayBiotech Inc., Norcross, GA, USA) was used to determine the cytokines in coculture experiments. The signal intensities of the cytokines were quantified using the Image J software (NIH Image, National Institutes of Health, USA). A fold change ≥ 3 after coculture was considered as potential cytokine candidate. The chemokine (C-X-C motif) ligand 1 (CXCL1), CXCL5, granulocyte-macrophage colony-stimulating factor (GM-CSF), interleukin-6 (IL-6), monocyte chemoattractant protein 3 (MCP-3) levels serum-free CM collected from coculture experiment of eMSCs with endometrial epithelial and stromal cells from the menstrual phase were determined using enzyme-linked immunosorbent assays (ELISA; IL-6, Invitrogen; CXCL1, CXCL5, GM-CSF and MCP-3, R&D Systems). Four candidate cytokines were shortlisted, and recombinant CXCL1 (1000 pg/ml; PeproTech, Rocky Hill, NJ, USA), CXCL5 (600 pg/ml; PeproTech), GM-CSF (500 pg/ml, PeproTech) and IL-6 (500 pg/ml, PeproTech) at concentrations found in the coculture condition was added to the growth medium of the eMSCs seeded at clonal density (500 cells/cm^2^) for 15 days.

### Gene silencing

EMSCs were plated in 48-well plates at a density of 8 × 10^3^/well in OptiMEM (Invitrogen) and the following day transfected with 10 pmol of siRNA directed against leucine-rich repeat-containing G-protein coupled receptor 5 (LGR5, ID s16275, Ambion) or random siRNA with scrambled sequence (Ambion) using Lipofectamine RNAiMax transfection reagent (Invitrogen) according to the manufacturer’s instructions. Twenty four hours after transfection, the medium was replaced with OptiMEM. The cells were then assayed using the WNT reporter system as described [9]. The knockdown efficiency was assessed by western blotting (Additional file: Fig. S1J).

### Statistical analysis

Data were analysed using the GraphPad PRISM software (version 5.00; GraphPad Software Inc., San Diego, CA, USA). Distribution normality was examined using the D’Agostino and Pearson test. Mann-Whitney test was performed to determine the statistical significance between the two groups. Kruskal-Wallis test followed by Dunn’s post-test were used for multiple group comparison. Data are presented as mean ± SEM. *P* < 0.05 was considered statistically significant.

## Results

### Endometrial cells from the menstrual phase promote clonogenicity and phenotypic expression of eMSCs

**Fig. 1:**
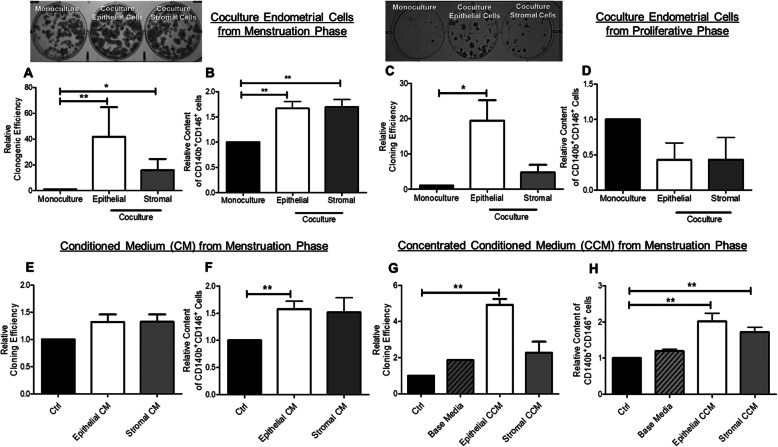
Coculture of eMSCs with niche cells from menstruation and proliferative phase. **a** Representative image showing the distribution of colonies in monoculture, epithelial coculture and stromal coculture. Relative cloning efficiency of eMSC colonies in monoculture, coculture with epithelial or stromal cells from menstrual phase (*n* = 8). **b** The relative percentage of CD140b^+^CD146^+^ cells (*n* = 8). **c** Relative cloning efficiency of eMSC colonies in monoculture, coculture with epithelial or stromal cells from proliferative phase (*n* = 5). **d** The relative percentage of CD140b^+^CD146^+^ cells (*n* = 5). **e** Relative cloning efficiency of eMSC colonies in growth medium (ctrl), epithelial and stromal CM (*n* = 10). **f** The relative proportion of CD140b^+^CD146^+^ cells after CM treatment (*n* = 10). **g** The effect of CCM from menstrual phase on eMSCs, relative cloning efficiency of eMSC colonies in growth medium (ctrl), base media, epithelial CCM and stromal CCM (*n* = 5). **h** Relative proportion of CD140b^+^CD146^+^ cells after treatment. All the data were normalized to the control. Results shown as mean ± SEM; *P < 0.05, **P < 0.01. CCM, concentrated conditioned medium; CM, conditioned medium; eMSCs, endometrial mesenchymal stem-like cells

To investigate the role of endometrial niche cells on clonogenic activity of eMSCs, the cells were cocultured in vitro. The expression of the eMSC surface markers (CD140b and CD146) was evaluated by flow cytometry. Coculture with endometrial cells from the menstrual phase increased the formation of CFUs when compared with those without coculture (monoculture control); the relative cloning efficiencies formed by eMSCs after coculture with the menstrual phase epithelial (41.70 ± 22.89 fold, *P* < 0.01) and stromal (15.83 ± 8.88 fold, *P* < 0.05) niche cells were significantly higher than that of monoculture (*n* = 8, Fig. 1a). The relative percentage of cells co-expressing CD140b and CD146 was significantly higher in the coculture groups from the menstrual phase (epithelial: 1.67 ± 0.14 fold, *P* < 0.01; stromal: 1.69 ± 0.15 fold, *P* < 0.01, *n* = 8, Fig. 1b) than that in the monoculture group.

To determine if the interactions of endometrial niche cells on eMSC maintenance was menstrual cycle-phase specific, epithelial and stromal cells from the proliferative phase were used in the coculture system. Although there was an increase of the relative cloning efficiency of eMSCs in coculture with epithelial cells (19.44 ± 5.81 fold) when compared with the monoculture (*P* < 0.05, *n* = 5, Fig. 1c), epithelial or stromal cells from the proliferative phase did not affect the phenotypic expression of the two eMSC markers (epithelial, 0.43 ± 0.24 fold; stromal, 0.43 ± 0.31 fold, *n* = 5, Fig. 1d).

The cell type specificity of the observed effect on eMSCs was evaluated using the human oviductal epithelial cells (OE-E6/E7) and the human foreskin fibroblasts (HFF-1). The relative cloning efficiency of eMSCs when coculture with OE-E6/E7 was significantly higher (*P* < 0.01), while those with HFF-1 only exhibited an increasing trend without reaching statistically significance (*n* = 5, Additional file: Fig. S1G). HFF-1 but not OE-E6/E7 coculture increased the co-expression of the eMSC markers when compared to the monoculture (*n* = 5, *P* < 0.05, Additional file: Fig. S1H).

### Conditioned medium from the menstrual phase endometrial epithelial and stromal cells increases phenotypic expression of eMSCs

To confirm that secretory products from the endometrial cells mediated the coculture effect, eMSCs were cultured in the menstrual phase CM. The relative cloning efficiency was similar after cultured in epithelial (1.32 ± 0.14 fold, *n* = 10) and stromal (1.32 ± 0.14 fold, Fig. 1e) CM when compared with the control. Epithelial CM increased the relative percentage of CD140b^+^CD146^+^ cells after treatment (1.58 ± 0.15 fold, *n* = 10, *P* < 0.01, Fig. 1f). However, the corresponding value for the stromal CM did not reach statistical significance, which might be due to high inter-patient variation (1.52 ± 0.27 fold, *P* > 0.05, Fig. 1f).

The lack of effect of CM on eMSC proliferation could be due to accumulation of endometrial cells derived metabolic waste that decreased proliferation during conditioning. Therefore, serum-free CM from the menstrual phase endometrial cells was collected and the metabolic waste in the CM was removed by ultrafiltration, which also concentrated the high molecular weight secretory factors derived from the endometrial cells (CCM). The base medium was also passed through the ultrafiltration unit to serve as the control. Supplementation of CCM from epithelial cells stimulated formation of the CFUs (4.06 ± 0.19 fold, *n* = 5, *P* < 0.01 Fig. 1g) and increased the co-expression of the CD140b and CD146 markers (2.01 ± 0.23 fold, *n* = 5, *P* < 0.01, Fig. 1h) when compared to that from the control. Addition of CCM from the stromal cells also significantly increased the co-expression of CD140b and CD146 (1.72 ± 0.14 fold, *P* < 0.01, Fig. 1h).

### The WNT/β-catenin signalling is involved in communication between eMSCs and endometrial cells from menstrual phase

**Fig. 2:**
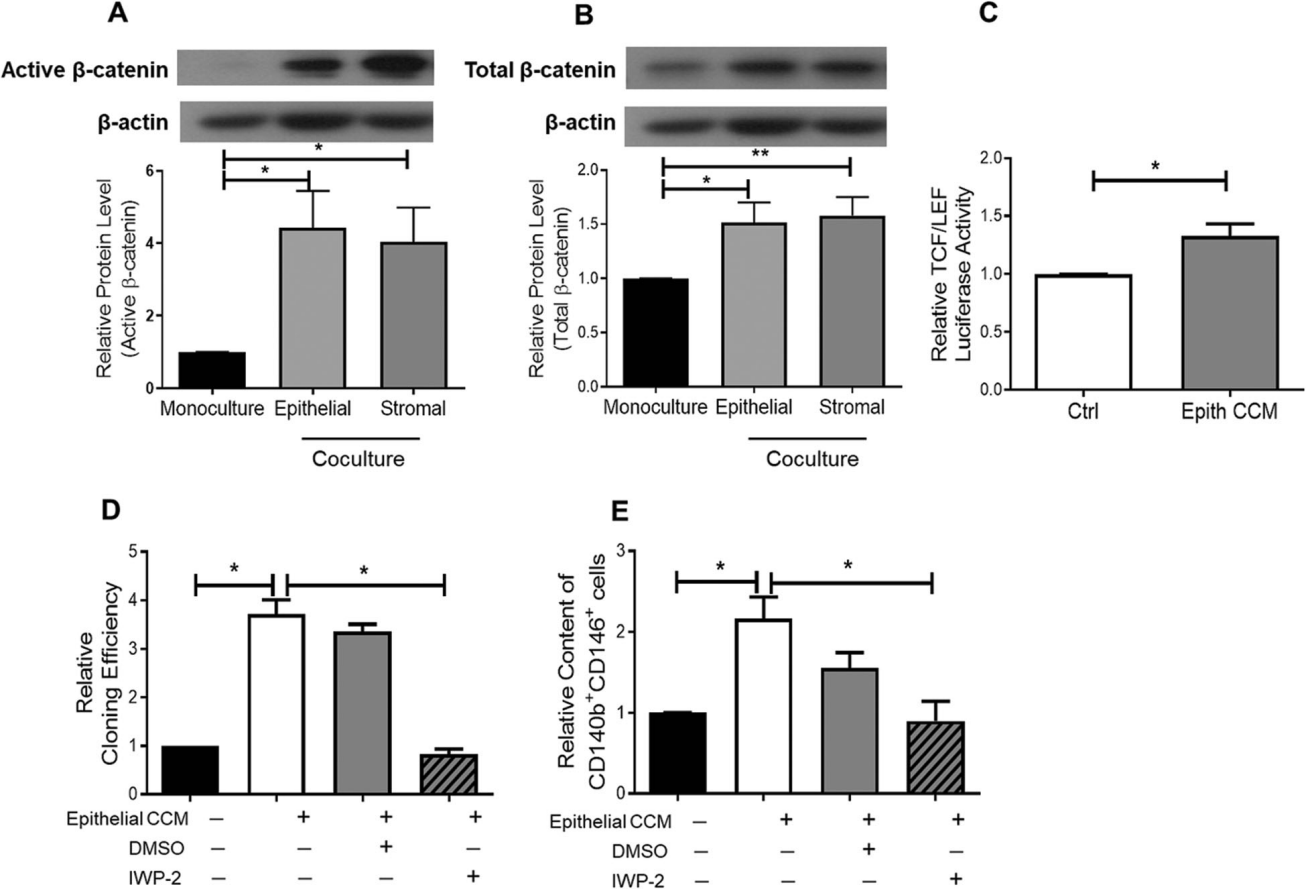
Expression of active and total β-catenin in eMSCs after coculture with niche cells from menstruation. The protein levels of **a** active β-catenin and **b** total β-catenin in monoculture, coculture with epithelial (*n* = 7) or stromal cells (*n* = 8) relative to β-actin. Representative western blotting bands of active β-catenin, total β-catenin and β-actin in eMSCs monoculture, coculture with epithelial or stromal cells from menstrual phase. **c** The TCF/LEF luciferase signal of eMSCs culture in growth medium (ctrl) and epithelial CCM from menstrual phase. Data were normalized to control (*n* = 10). **d** Relative cloning efficiency of eMSCs colonies in growth medium, epithelial CCM from menstrual phase, epithelial cell CCM with DMSO and epithelial cell CCM with IWP-2 at 1.25 μM (*n* = 4). **e** Relative proportion of CD140b^+^CD146^+^ cells after treatment. Results are shown as mean ± SEM; *P < 0.05, **P < 0.01. CCM, concentrated conditioned medium; eMSCs, endometrial mesenchymal stem-like cells

The WNT signalling plays a role in population expansion and self-renewal of adult stem cells in various mammalian tissues [6] including endometrium [10]. Therefore, the protein expression of active and total β-catenin in eMSCs after coculture with endometrial cells was evaluated. Coculture with epithelial or stromal cells from the menstrual phase significantly increased the relative expression of active β-catenin (epithelial: 4.43 ± 1.02 fold, *P* < 0.05; stromal: 4.03 ± 0.95 fold, *n* = 7, *P* < 0.05; Fig. 2a) and total β-catenin (epithelial: 1.51 ± 0.19 fold, *P* < 0.05; stromal, 1.58 ± 0.17 fold, *n* = 7, *P* < 0.01, Fig. 2b) when compared to the monoculture. Luciferase assay for TCF/LEF transcriptional activity was used to determine activation of WNT/β-catenin signalling after treatment with menstrual epithelial CCM. Consistently, the treatment significantly increased the TCF/LEF transcriptional activity of eMSCs by 1.33 ± 0.11 fold when compared to control (*n* = 10, *P* < 0.05, Fig. 2c).

In order to determine the importance of WNTs for the observed effects, we tested the impact of blocking WNT secretion using IWP-2. Since epithelial CCM supported formation of CFUs and phenotypic expression of eMSCs to a higher extent than stromal CCM (Fig. 1g, h), we used epithelial CCM for the subsequent functional assays. The addition of epithelial CCM significantly increased the formation of CFUs and percentage of CD140b^+^CD146^+^ cells by 3.06 ± 0.30 fold (*n* = 5, *P* < 0.05, Fig. 2d) and 2.16 ± 0.27 fold (*n* = 5, *P* < 0.05, Fig. 2e), respectively when compared to the control. After treatment of the epithelial cells with IWP-2, CCM from the treated cells lost the ability to enhance clonogenicity (Fig. 2d) and phenotypic marker expression (Fig. 2e).

### eMSCs express functional LGR5

**Fig. 3:**
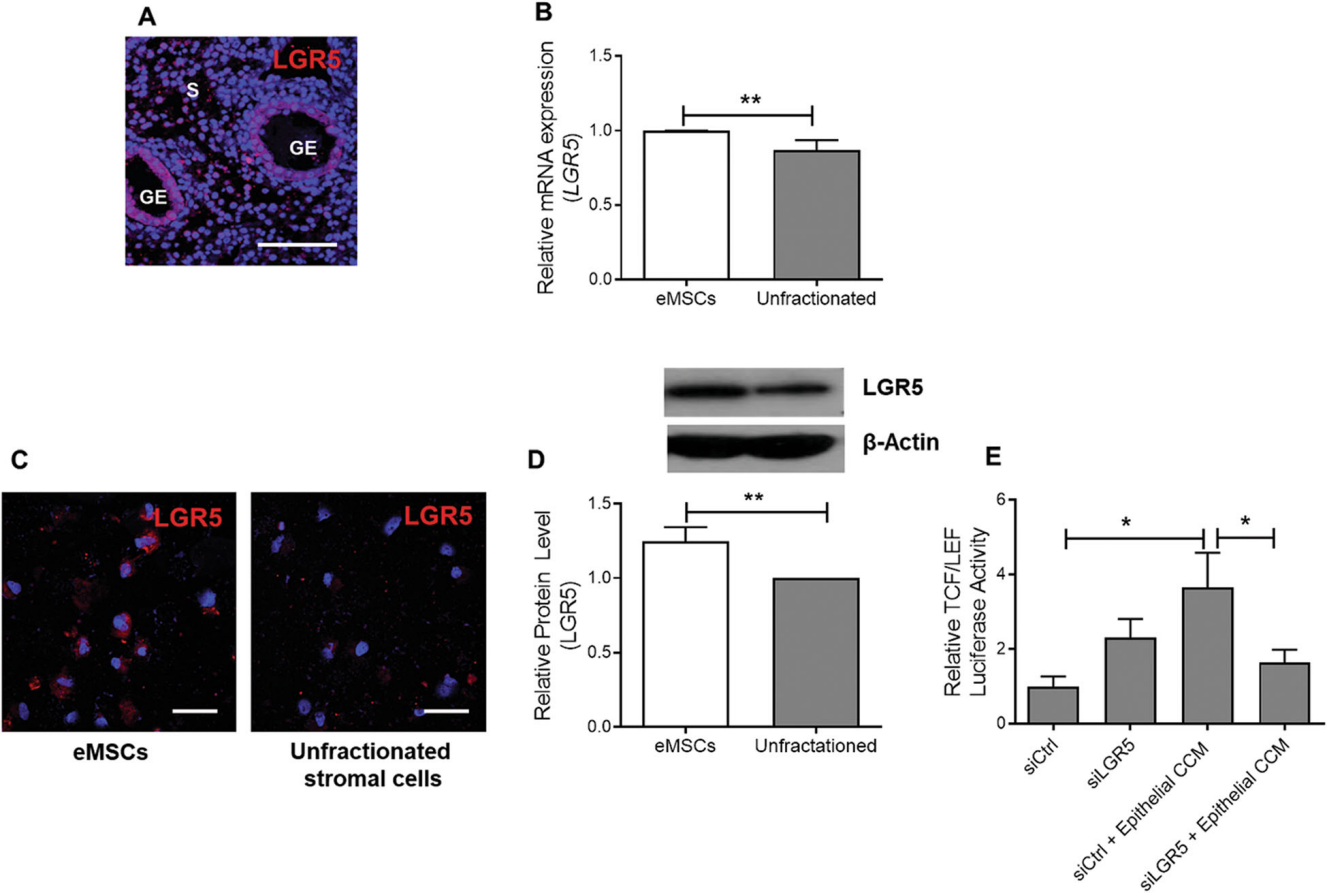
Expression of LGR5 in eMSCs and endometrial stromal cells. **a** Immunofluorescent staining of LGR5 (red) in human endometrium. **b** The relative gene expression (*n* = 9) and **c** immunofluorescent images of LGR5 in eMSCs and unfractionated endometrial stromal cells. **d** Representative western blotting image and quantitative analysis of LGR5 expression in eMSCs and unfractionated endometrial stromal cells. (*n* = 6). **e** The TCF/LEF luciferase signal of eMSC after transfection with siRNA directed towards LGR5 (siLGR5), after transfection with scrambled control siRNA (siCtrl), culture with epithelial CCM from the menstrual phase after transfection with siCtrl and culture with epithelial CCM after transfection with siLGR5 (*n* = 8). Results are shown as mean ± SEM; *P < 0.05, ** *P* < 0.01. Scale bar, 100 μM. CCM, concentrated conditioned medium; eMSCs, endometrial mesenchymal stem-like cells; GE, glandular epithelium; LGR5, leucine-rich repeat-containing G-protein coupled receptor 5; s, stroma; siLGR5, siRNA to LGR5; siCtrl, scrambled control siRNA

LGR5, a well-known marker of epithelial stem cells, is expressed in the perivascular region of endometrial stroma where the CD140b^+^CD146^+^ cells reside. LGR5 interacts with secreted R-spondins to modulate WNT signal strength on WNT-responsive stem cells in multiple tissues [20]. In human endometrium, the LGR5 expression was higher in glandular epithelial cells compared with stromal cells (Fig. 3a). We compared the expression of LGR5 in unfractionated endometrial stromal cells and eMSCs of the same patient and found higher mRNA expression of *LGR5* in the latter than in the former (*n* = 9, *P* < 0.01, Fig. 3b). Consistently, the protein expression of LGR5 using immunofluorescence (Fig. 3c) and western blotting (*n* = 5, *P* < 0.01, Fig. 3d) was more abundant in eMSCs than the unfractionated stromal cells. To determine the role of LGR5 in menstrual phase epithelial CCM induced WNT signalling, the expression of LGR5 was knocked down using LGR5-siRNA. As expected, the epithelial CCM-induced increase TCF/LEF luciferase activity was abolished upon treatment of eMSCs with LGR5-siRNA (*n* = 8, *P* < 0.05, Fig. 3e).

### RSPO1 potentiates the action of WNT3A on self-renewal of eMSCs

**Fig. 4:**
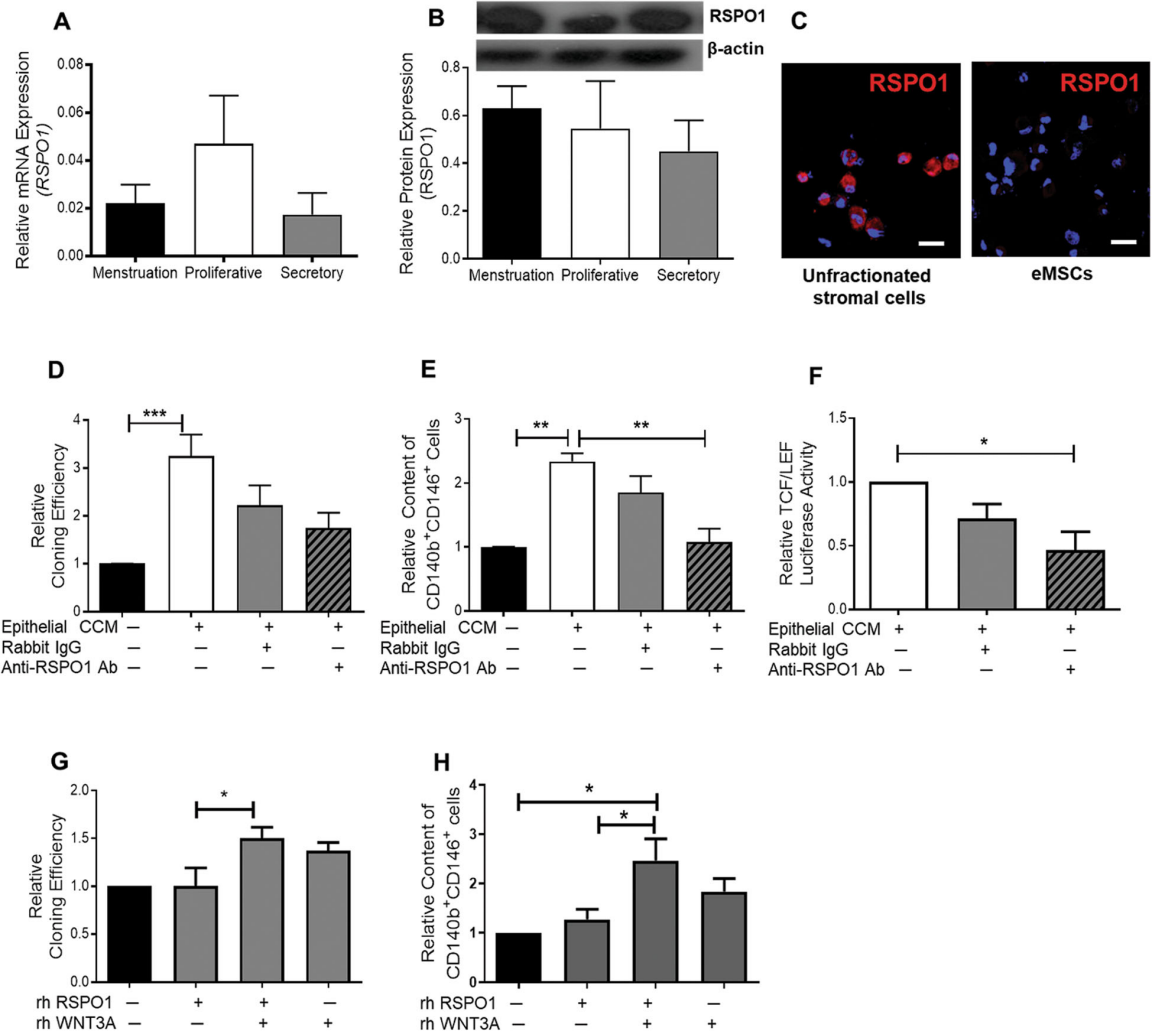
The role of RSPO1 on eMSC clonogenicity and phenotypic expression. **a** Gene expression of *RSPO1* in stromal cells from the menstrual, proliferative and secretory phase using qPCR (*n* = 7). **b** Protein expression of RSPO1 in stromal cells from the menstrual, proliferative and secretory phase using western blotting. (*n* = 7). **c** Representative immunofluorescent images showing unfractionated endometrial stromal cells and eMSCs (CD140b^+^CD146^+^cells) expressing RSPO1 (red). **d** Relative cloning efficiency of eMSC colonies in growth medium, epithelial cell CCM from the menstrual phase, epithelial cell CCM with addition of rabbit IgG and epithelial cell CCM with addition of RSPO1 antibody at 1 μg/ml (*n* = 7). **e** Relative proportion of CD140b^+^CD146^+^ cells after treatment (*n* = 7). Data were normalized to control. **f** The TCF/LEF luciferase signal of eMSCs with epithelial CCM from the menstrual phase; epithelial cell CCM with addition of rabbit IgG and epithelial CCM with addition of human anti-RSPO1 antibody at 1 μg/ml. Data normalized to growth medium (*n* = 5). **g** Relative cloning efficiency of eMSC colonies in growth medium, 50 ng/ml of rhRSPO1, 50 ng/ml of rhRSPO1 + 25 ng/ml of rhWNT3A and 25 ng/ml of rhWNT3A alone (*n* = 7). **h** Relative proportion of CD140b^+^CD146^+^ cells after treatment (*n* = 7). Results are shown as mean ± SEM; *P < 0.05, **P < 0.01, *** *P* < 0.001. Scale bar, 50 μM. CCM, concentrated conditioned medium; eMSCs, endometrial mesenchymal stem-like cells; rh, recombinant human

Next, we investigated whether the canonical WNT ligands together with R-spondin can synergize the WNT/β-catenin pathway, enhancing the WNT signalling [21]. The mRNA (Fig. 4a) and protein (Fig. 4b) expression of RSPO1 in stromal cells was similar across the menstrual cycle. Strong immunofluorescence signals of RSPO1 (Fig. 4c) were detected in the unfractionated stromal cells when compared to that in eMSCs. The addition of anti-RSPO1 antibody reduced the stimulatory actions of epithelial CCM on the relative cloning efficiency (*n* = 7, Fig. 4d) and the expression of eMSC markers (*n* = 7, Fig. 4e). The neutralization antibody also significantly reduced the TCF/LEF luciferase activity of eMSCs by 0.55 ± 0.09 fold (*P* < 0.05, *n* = 5, Fig. 4f) when compared with those treated with the epithelial CCM.

Treatment of recombinant RSPO1 protein alone had no effect on colony formation and phenotypic expression of eMSCs (Fig. 4g, h). WNT3A dose-dependently enhanced the phenotypic expression of eMSCs. R-spondins act on LGR receptor and stabilize frizzled receptors to potentiate WNT signalling [22, 23]. To test the potentiating action, recombinant RSPO1 was used to treat eMSCs in the presence of recombinant WNT3A at 25 ng/ml, which is just insufficient to enhance the proportion of CD140b^+^CD146^+^ cells (Additional file: Fig. S1I). In such condition, combined RSPO1 and WNT3A but not their individual alone treatment increased in the proportion of CD140b^+^CD146^+^ cells (2.46 ± 0.44 fold) when compared to treatment with RSPO1 protein alone (1.27 ± 0.21 fold, *P* < 0.05) and control (*n* = 7, *P* < 0.05, Fig. 4h). The combined treatment also significantly increased the clonogenicity (1.50 ± 0.19 fold, *n* = 7, *P* < 0.05, Fig. 4g) when compared to the control.

### The role of cytokines in the regulation of eMSCs at menstruation

**Fig. 5:**
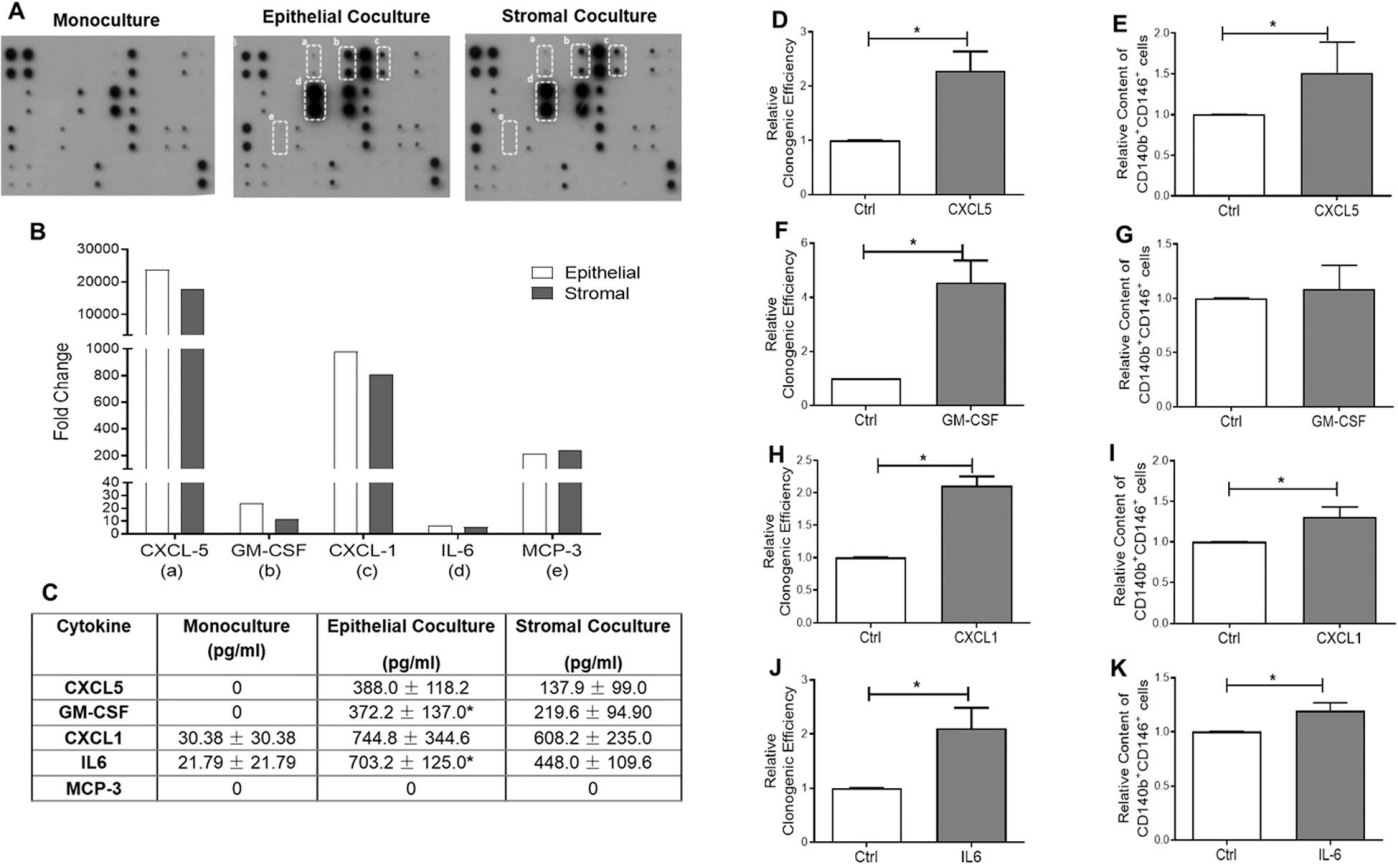
Expression of cytokines in menstrual conditioned medium. **a** Cytokine array showing the densitometry spots of serum-free conditioned medium from monoculture, coculture with epithelial or stromal cells from the menstrual phase. **b** Fold change of cytokines: CXCL5, GM-CSF, CXCL-1, IL-6 and MCP-3 in conditioned medium coculture with epithelial (white bars) or stromal (grey bars) cells from the menstrual phase (*n* = 2). **c** Table showing the concentration of cytokines in monoculture, coculture with epithelial or stromal cells from the menstrual phase (*n* = 3). Relative cloning efficiency (*n* = 4) and proportion of CD140b^+^CD146^+^ cells (*n* = 4) in growth medium (ctrl) or addition of **d**, **e** CXCL5 at 600 pg/ml, **f**, **g** GM-CSF at 500 pg/ml, **h**, **i** CXCL1 at 1000 pg/ml and **j**, **k** IL-6 at 500 pg/ml. Results are shown as mean ± SEM; *P < 0.05. CXCL1, C-X-C motif ligand 1; CXCL5, C-X-C motif ligand 5; IL-6, interleukin 6; GM-CSF, granulocyte-macrophage colony-stimulating factor; MCP-3, monocyte chemotactic protein-3

To elucidate the other potential regulators of eMSCs during menstruation, the role of cytokines and chemokines from niche cells on eMSC maintenance was examined. The cytokine expression profile in monoculture and coculture with menstrual epithelial or stromal cells was determined by the cytokine array. Densitometric analysis comparing monoculture with coculture of menstrual epithelial and stromal cells revealed increases of several cytokines by more than 3-fold (*n* = 2, Fig. 5a). These cytokines were CXCL5 (epithelial, mean: 23,847-fold; stromal, 17,916-fold), GM-CSF (epithelial, 24-fold; stromal, 12-fold), CXCL1 (epithelial, 981-fold; stromal, 809-fold), IL-6 (epithelial, 6-fold; stromal, 5-fold) and MCP-3 (epithelial, 212-fold; stromal, 243-fold) (Fig. 5b). Next, ELISA was used to validate the cytokines identified in the CM. The amount of GM-CSF and IL-6 was significantly elevated after coculture of menstrual epithelial cells when compared to monoculture (*n* = 3, Fig. 5c). There was no difference for CXCL5 or CXCL1 and the level of MCP3 was undetectable. The functional effect of the four candidate cytokines were assessed with clonogenic assay and flow cytometry. Addition of CXCL5 (Fig. 5d, e), CXCLl (Fig. 5h, i) and IL-6 (Fig. 5j, k) significantly increased the colony formation (*n* = 4, *P* < 0.05) and phenotypic expression of eMSCs (*n* = 4, *P* < 0.05) when compared to control. GM-CSF could only increase the clonogenic activity of eMSCs (*n* = 4, *P* < 0.05, Fig. 5f), while it did not affect the phenotypic expression (*n* = 4, Fig. 5g).

## Discussion

Endometrial stromal and epithelial cell interactions undergo phasic changes across the reproductive cycle [24]. The changes in hormonal milieu and tissue microenvironment introduce distinct environmental cues and impose significant demand on acute cellular responses to maintain tissue homeostasis. An in vitro approach to replicate an in vivo microenvironment is coculture of stem/progenitor cells with endometrial cells. The dynamic regenerative characteristic displayed by the human endometrium indicates there is a fine balance between stem cell self-renewal and differentiation. Little is known about the local niche signals for eMSC self-renewal. Endometrial regeneration begins at menstruation; therefore, it was hypothesized that the niche cells of eMSCs provide a specialized microenvironment during menstruation activating the stem cells to restore the dynamic endometrial lining.

In this study, we found that epithelial and stromal cells from menstruation samples stimulated eMSCs proliferation. The increase proportion of CD140b^+^CD146^+^ cells after coculture demonstrated that the niche cells supported self-renewal of eMSCs. The comparison of eMSCs from monoculture and coculture indicated a role of WNT/β-catenin signalling in regulating the activities of eMSCs during menstruation. To our knowledge, this is the first report studying the effect of menstrual niche cells on human eMSCs; the niche cells at menstruation promote eMSC expansion by symmetric division to sustain the stem cell pool during endometrial regeneration. Findings from our previous study support the present observations by demonstrating a higher proportion of eMSCs in the menstrual phase undergoing more rounds of self-renewal [5].

Niche cells isolated from the proliferative phase did not display the same phenomenon. The observed declining trend in the proportion of CD140b^+^CD146^+^ cells when cocultured with the proliferative phase niche cells suggested that the stem cells are undergoing differentiation in such scenario. Hence, the maintenance of eMSCs is “phase-specific.” The effect of secretory phase niche cells on eMSCs was not assessed due to the coordination of sample availability during the period for the coculture studies. We speculate that these niche cells are unlikely to support the eMSC population since the self-renewal activity of eMSCs at the secretory phase is lower than those at the menstrual phase [5].

The endometrial cells were separated into epithelial and stromal fractions to delineate the cell type that can interact with eMSCs. Both epithelial and stromal cells can promote eMSC proliferation and self-renewal by providing soluble secretory factors. The cell type specificities of maintaining eMSCs were studied using the cell types of different origins. Although OE E6/E7 cells stimulated the formation of the CFUs, these cells could not sustain the proportion of CD140b^+^CD146^+^ cells suggesting that the oviductal cells do not secrete factors similar to that of the endometrial niche cells. Coculture with the HFF-1 cells increased the phenotypic expression of eMSCs. This finding was unexpected, since these cells are not at proximal anatomic location of the eMSCs in vivo. It is possible that HFF-1 cell may coincidently provide secretory products which can support the self-renewal of the eMSCs. One possible factor is interleukin-6 [25], which is also produced by endometrial niche cells and has a stimulatory action on stem cell renewal (see below).

In contrast to the coculture system, the formation of CFUs did not change after inclusion of either the epithelial or the stromal CM. Several possibilities may lead to the differences observed between coculture and CM studies. First, there may be a two-way communication between the niche cells and the eMSCs in the coculture system; not only the eMSCs receive signals from the niche cells, the niche cells can also respond to the secretory molecules from the eMSCs. This reciprocal communication can alter the behaviours of both cell types. Thus, the niche cells are supporting the eMSCs, while the eMSCs are stimulating the niche cells to secrete factors important for the stemness of eMSCs. Second, niche cells derived secretory products are continuously produced in a coculture system. The CM was collected only after a 48 h of conditioning. Hence, the amount of secretory products collected could be limited. Third, the niche cells deplete the nutrient in and excrete metabolic waste to the CM, making the CM less favourable for the maintenance of eMSCs.

In order to remove the possible influence of depletion of nutrient and accumulation of metabolic waste in the CM, the high molecular weight (> 10 kDa) secretory factors of the niche cells were enriched. Serum-free base medium was used to acquire purified secreted factors from niche cells without the influence of the growth factors from the serum supplement. Overall, the clonogenic activity increased after addition of the concentrated epithelial CM. The increase in eMSC phenotype was consistent with the CM treatment confirming the ability of epithelial cells from menstruating endometrium in stimulating the self-renewal of eMSCs.

Since the stimulatory effect of endometrial niche cells did not require direct contact with eMSCs, we examined two mechanisms of actions of endometrial niches cells, namely via WNT ligands and cytokines or chemokines. We have previously demonstrated the involvement of WNT signalling in endometrial stem cells renewal [9, 10]. An elevation of total and active β-catenin in the eMSCs after coculture with menstrual phase endometrial cells confirmed the involvement of the WNT/β-catenin signalling in eMSCs regulation. This study further demonstrated that the R-spondin/LGR signalling facilitates the actions of WNT ligands on self-renewal of eMSCs.

LGR5 is a well-known marker of epithelial stem cells [26]. Immunoreactive LGR5 has been localized to the perivascular region of the stroma of human endometrium [27], where the CD140b^+^CD146^+^ cells reside. The LGR5 mRNA is stably expressed throughout the menstrual cycle [28]. Here, we confirmed that the eMSCs expressed LGR5 immunoreactivities. In addition, knockdown experiment and TCF/LEF reporter assay demonstrated that the LGR5 in eMSCs in functional in regulating WNT signalling. The ligand of LGR5 is R-spondins. The R-spondin/LGR signalling fine-tunes the WNT pathway output [29]. RSPO1 is required for the formation of endometrial epithelial organoids [30, 31]. We showed higher RSPO1 protein expression in the unfractionated stromal than the eMSCs, and that RSPO1 potentiates the action of WNT3A on self-renewal of eMSCs, probably by stabilization of frizzled receptors through R-spondin/LGR interaction [22, 23].

The cytokine array comparison of the monoculture and coculture CM uncovered several candidate cytokines/chemokines. Subsequent ELISA results confirmed that their production by the eMSCs and the menstruation niche cells. Overall, the cytokine/chemokines levels in CM were higher in coculture than in monoculture. It is likely that most of the cytokine/chemokines were derived from the niche cells because their cell numbers were 80-fold higher than that of eMSCs in coculture.

The increase of clonogenicity and proportion of eMSCs after treatment suggest that CXCL1, CXCL5 and IL-6 may be potential regulators of eMSCs. The production of CXCL1 and CXCL5 in the endometrium has been well studied. Both the endometrial epithelial and stromal cells produce CXCL5 upon stimulation by other cytokines [32]. CXCL1 is expressed in the endometrial stroma [33]. Under- or over-expression of these chemokines have been linked to major events in the endometrium such as implantation and endometriosis [34]. However, their role in endometrial repair remains unknown. CXCL5 is known to be essential for bone marrow mesenchymal stem cell (BMSC) invasion and migration [35]. Whether CXCL5 has a similar effect on eMSCs requires more detailed investigation.

IL-6 is essential in maintaining the BMSC stemness through the ERK1/2 signalling pathway [36]. The expression of IL-6 is significantly higher in the undifferentiated BMSCs and decreases dramatically during osteogenic differentiation. Moreover, BMSCs exhibit their immunomodulatory effect through cytokines [37]. Given the importance of IL-6 on MSCs, it is not surprising that this cytokine had a positive effect on eMSCs. Our preliminary data suggest IL-6 is a potential regulator on eMSCs activation after endometrial breakdown; it increases clonogenicity and phenotypic expression of eMSCs. In humans, abnormal level of IL-6 has been linked to many disorders of endometrium. Retrograde menstruation is one of the possible causes of endometriosis [38]. The peritoneal fluid of women with endometriosis has increased IL-6 levels [39]. It is possible that the stem/progenitor cells present in the menstrual effluent can respond to the high level of IL-6 in the peritoneal cavity, contributing to the development of endometriotic lesions at ectopic sites. How does IL-6 modulate eMSC activities remains to be investigated. It is interesting to note that IL-6 activates Wnt pathway of rat mesenchymal stem cells via JAK2/STAT3 signalling [40]. In mice, IL-6 regulates gut epithelial crypt homeostasis through the Wnt signalling pathway [41]. In humans, there is a positive feedback loop between IL-6 and WNT5A in melanoma cells [42], and WNT5A-FZD4/LRP5 signalling supports self-renewal of embryonic stem cells [43] and eMSCs [9].

Shedding and repair of the functional endometrial layers occur simultaneously during menstruation [44]. Such rapid regenerative process indicates the presence of signals for stem cell activation during menstruation. Our findings demonstrated two niche signals regulating eMSC activities during menstruation, namely WNT ligand/RSPO1 signalling and cytokine/chemokine signalling. A better understanding of the signals within the uterine microenvironment during endometrial repair will unravel new concepts in dissecting the responses of self-renewing cells to defined factors in vitro. The findings will be useful for recreating the uterine microenvironment and tissue engineering application to treat women with disorders associated with inadequate endometrium such as Asherman’s syndrome.

## Conclusion

Our findings indicate a role of WNT/β-catenin signalling in regulating activities of endometrial stem/progenitor cells during menstruation. Certain cytokines at menstruation can stimulate the proliferation and self-renewal activities of eMSCs. Understanding the mechanism in the regulation of eMSCs may contribute to treatments of endometrial proliferative disorders.

## Supplementary information

**Additional file 1: Figure S1.** Expression of eMSC surface markers (CD140b & CD146) in clonally derived cells. Representative figures showing the gating strategy to evaluate the phenotypic markers of eMSCs (CD140b^+^CD146^+^ cells) using flow cytometry. **(A)** Clonally derived cells were gated on flow cytometric profile based on the forward scatter (FSC, associated with cell size) and side scatter (SSC, associated with cell granularity). **(B)** Single cells were separated from doublets and aggregated cells based on their SSC area (SSC-A) and SSC height (SSC-H) on the dot plot. **(C)** Single parameter histograms for individual markers: CD140b-PE^+^ cells and, CD146-FITC^+^ cells. Grey area indicates background fluorescence with isotype matched IgG control. The percentage of cell maintaining CD140b-PE^+^ and CD146-FITC^+^ on the upper right quadrant of the dot plot from **(D)** monoculture, **(E)** coculture with epithelial niche cells from the menstrual phase, **(F)** coculture with stromal niche cells from the menstrual phase. **(G)** Relative cloning efficiency eMSC colonies. in monoculture, coculture with OE E6/E7 or HFF-1 (*n* = 5). **(H)** Relative proportion of CD140b^+^CD146^+^ cells after coculture (*n* = 5). Data normalized to the monoculture group. **(I)** Relative proportion of CD140b^+^CD146^+^ cells in ctrl (white bar) and 12.5, 25, 50 ng/ml of rhWNT3A (grey bars). **(J)** Western blotting image and quantitative analysis of LGR5 expression in stromal cells after gene silencing with si-RNA (*n* = 3). Results are shown as mean ± SEM; *P < 0.05, **P < 0.01. Abbreviations: eMSCs, endometrial mesenchymal stem-like cells; HFF, human foreskin fibroblasts, OE E6/E7, oviductal epithelial cells, rh, recombinant human.

## Data Availability

The data that support the findings of this study are available from the corresponding author upon reasonable request.

## Abbreviations

CM: Conditioned medium
CCM: Concentrated conditioned medium
CFUs: Colony forming units
CXCL1: C-X-C motif ligand 1
CXCL5: C-X-C motif ligand 5
eMSCs: Endometrial mesenchymal stem-like cells
GM-CSF: Granulocyte-macrophage colony-stimulating factor
IL-6: Interleukin 6
LGR5: Leucine-rich repeat-containing G-protein coupled receptor 5
MCP-3: Monocyte chemotactic protein-3
RSPO1: R-spondin-1
TCF/LEF: T cell factor/lymphoid enhancer-binding factor
