# Correction to: Understanding the regulatory mechanisms of endometrial cells on activities of endometrial mesenchymal stem-like cells during menstruation

**DOI:** 10.1186/s13287-022-02871-7

**Published:** 2022-05-15

**Authors:** Shan Xu, Rachel W. S. Chan, Tianqi Li, Ernest H. Y. Ng, William S. B. Yeung

**Affiliations:** 1 Department of Obstetrics and Gynaecology, Second Affiliated Hospital of Xi’an Jiaotong University, Xi’an, Shaanxi China; 2 Department of Obstetrics and Gynaecology, LKS Faculty of Medicine, The University of Hong Kong, Pokfulam, Hong Kong, SAR China; 3 Shenzhen Key Laboratory of Fertility Regulation, Reproductive Medicine Centre, The University of Hong Kong Shenzhen Hospital, Shenzhen, Guangdong China

## Correction to: Xu et al. Stem Cell Research & Therapy (2020) 11:239 10.1186/s13287-020-01750-3

The original article [1] noted two erroneous grant numbers which have both since been corrected.

