# Supplementary material for: Understanding the regulatory mechanisms of endometrial cells on activities of endometrial mesenchymal stem-like cells during menstruation
Source: Stem Cell Res Ther. 2020 Jun 17;11:239. doi: 10.1186/s13287-020-01750-3 (PMC7302161; doi:10.1186/s13287-020-01750-3)
Supplement: Supplementary file 1 — Additional file 1: Figure S1. Expression of eMSC surface markers (CD140b & CD146) in clonally derived cells. Representative figures showing the gating strategy to evaluate the phenotypic markers of eMSCs (CD140b+CD146+ cells) using flow cytometry. (A) Clonally derived cells were gated on flow cytometric profile based on the forward scatter (FSC, associated with cell size) and side scatter (SSC, associated with cell granularity). (B) Single cells were separated from doublets and aggregated cells based on their SSC area (SSC-A) and SSC height (SSC-H) on the dot plot. (C) Single parameter histograms for individual markers: CD140b-PE+ cells and, CD146-FITC+ cells. Grey area indicates background fluorescence with isotype matched IgG control. The percentage of cell maintaining CD140b-PE+ and CD146-FITC+ on the upper right quadrant of the dot plot from (D) monoculture, (E) coculture with epithelial niche cells from the menstrual phase, (F) coculture with stromal niche cells from the menstrual phase. (G) Relative cloning efficiency eMSC colonies. in monoculture, coculture with OE E6/E7 or HFF-1 (n = 5). (H) Relative proportion of CD140b+CD146+ cells after coculture (n = 5). Data normalized to the monoculture group. (I) Relative proportion of CD140b+CD146+ cells in ctrl (white bar) and 12.5, 25, 50 ng/ml of rhWNT3A (grey bars). (J) Western blotting image and quantitative analysis of LGR5 expression in stromal cells after gene silencing with si-RNA (n = 3). Results are shown as mean ± SEM; *P < 0.05, **P < 0.01. Abbreviations: eMSCs, endometrial mesenchymal stem-like cells; HFF, human foreskin fibroblasts, OE E6/E7, oviductal epithelial cells, rh, recombinant human. [file 13287_2020_1750_MOESM1_ESM.docx]

**Supplementary Table S1** - Age of women, menstrual phase and reason of hysterectomy in those who donated full thickness endometrial samples.

|  | Age | Menstrual Phase | Pathology |
| --- | --- | --- | --- |
| 1 | 41 | Proliferative | adenomyosis + leiomyomas |
| 2 | 45 | Proliferative | adenomyosis + leiomyomas |
| 3 | 40 | Proliferative | leiomyomas |
| 4 | 48 | Proliferative | adenomyosis + leiomyomas |
| 5 | 49 | Proliferative | adenomyosis + leiomyomas |
| 6 | 41 | Proliferative | leiomyomas |
| 7 | 49 | Proliferative | Leiomyomas |
| 8 | 48 | Proliferative | leiomyomas |
| 9 | 45 | Proliferative | leiomyomas |
| 10 | 42 | Secretory | leiomyomas |
| 11 | 46 | Secretory | leiomyomas |
| 12 | **52** | Secretory | leiomyomas |
| 13 | 49 | Secretory | leiomyomas |
| 14 | 45 | Secretory | leiomyomas |
| 15 | 50 | Secretory | adenomyosis + leiomyomas |
| 16 | 45 | Secretory | leiomyomas |
| 17 | 51 | Secretory | leiomyomas |
| 18 | 43 | Secretory | leiomyomas |
| 19 | 45 | Secretory | leiomyomas |
| 20 | 43 | Secretory | adenomyosis + leiomyomas |
| 21 | 47 | Secretory | leiomyomas |
| 22 | 48 | Secretory | adenomyosis |

**Supplementary Table S2** – Age of women and infertility cause in those who donated menstrual samples.

|  | Age |  |
| --- | --- | --- |
| 1 | 39 | Menstrual |
| 2 | 40 | Menstrual |
| 3 | 32 | Menstrual |
| 4 | 32 | Menstrual |
| 5 | 33 | Menstrual |
| 6 | 36 | Menstrual |
| 7 | 34 | Menstrual |
| 8 | 33 | Menstrual |
| 9 | 34 | Menstrual |
| 10 | 35 | Menstrual |
| 11 | 33 | Menstrual |
| 12 | 37 | Menstrual |
| 13 | 36 | Menstrual |
| 14 | 35 | Menstrual |
| 15 | 35 | Menstrual |
| 16 | 38 | Menstrual |
| 17 | 49 | Menstrual |
| 18 | 32 | Menstrual |
| 19 | 40 | Menstrual |
| 20 | 53 | Menstrual |
| 21 | 40 | Menstrual |
| 22 | 36 | Menstrual |
| 23 | 32 | Menstrual |

**Supplementary Table S3 - List of primary and secondary antibodies used for western blotting**

| **Primary Antibodies (WB)** | **Isotype** | **Dilution** | **Source** |
| --- | --- | --- | --- |
| **Active β-catenin:** mouse monoclonal active beta catenin; clone 8E7. | Mouse IgG1 | 1:500 | EMD Millipore |
| **LGR5:** rabbit polyclonal LGR5. | Rabbit IgG | 1:500 | Abcam |
| **β-catenin**: mouse monoclonal total beta catenin. | Mouse IgG | 1:1000 | BD Bioscience |
| **β-actin**: mouse monoclonal beta actin; clone AC-15. | Mouse IgG1 | 1:10000 | Sigma-Aldrich |

| **Secondary Antibodies (WB)** | **Dilution** | **Source** |
| --- | --- | --- |
| Mouse horseradish peroxidase | 1:10000 | GE Healthcare |
| Rabbit horseradish peroxidase | 1:10000 | GE Healthcare |

**Supplementary Table S4- List of primary antibodies used for immunofluorescent staining.**

| **Primary Antibodies (IF)** | **Dilution** | **Source** |
| --- | --- | --- |
| **LGR5:** rabbit polyclonal to LGR5. | 1:100 | Abcam |
| **RSPO1:** rabbit polyclonal to Rspondin-1 | 1:200 | Abcam |
